# Editorial: Unraveling the role of the liver myeloid compartment during hepatitis C virus cure

**DOI:** 10.1016/j.jhep.2023.04.016

**Published:** 2023-04-21

**Authors:** Emilie Crouchet, Thomas F. Baumert

**Affiliations:** 1 Université de Strasbourg, Inserm, Institut de Recherche sur les Maladies Virales et Hepatiques UMR_S1110, Strasbourg; 2 Service d’hépato-gastroentérologie, Hôpitaux Universitaires de Strasbourg, Strasbourg; 3 Institut hospitalo-universitaire (IHU), Université de Strasbourg, Strasbourg; 4 Institut Universitaire de France (IUF), Paris

**Keywords:** Direct-acting antivirals, exhaustion, innate immunity, interferon-stimulated genes, single cell RNA-Seq

Approval of direct-acting antivirals (DAAs) has revolutionized the treatment of chronic hepatitis C virus (HCV) infection by allowing elimination of the virus in more than 90% of infected patients without major adverse effects. Indeed, DAAs are the first curative therapeutic strategy for a chronic viral infection in humans [1]. Nevertheless, HCV infection remains a major risk factor for liver disease and cancer [2]. Several challenges remain, and a vaccine to prevent HCV infection is not available. DAA treatment does not prevent HCV re-infection in at-risk patients which remains an important challenge in drug abusers in the persisting opioid crisis [3,4]. Chronic HCV infection induces epigenetic modifications in the infected liver which can persist after DAA cure and are associated with risk to develop hepatocellular carcinoma (HCC) [5–7]. Moreover, recent studies have shown that persistent virus-induced host alterations also include perturbations of hepatic innate immunity and reprogramming of immune cells [8,9].

During infection, the virus is sensed by the patient host cells triggering innate immune response including activation/recruitment of myeloid cells. Myeloid cells comprise diverse populations of granulocytic and phagocytic leucocytes (i.e neutrophils, eosinophils, monocytes/macrophages, dendritic cells, mast cells) to detect pathogens and/or cells damaged by viral infections. Myeloid cells trigger immune responses by cytokines inducing inflammation and regulating the activation of adaptive immune responses. Moreover, myeloid cells have been shown an important immunoregulatory role not only in viral infection but also in inflammation, fibrosis and cancer [10,11].

During HCV infection, myeloid cells have been suggested to regulate both innate and adaptive anti-viral immune responses [12]. Interferon (IFN) responses are also crucial for viral cure, as demonstrated e.g. by the association of *IFNλ* polymorphism and HCV elimination. However, in most cases, HCV is able to evade immune response and the virus persists in association with increased interferon stimulated gene (ISG) levels in the liver [13]. Several studies have demonstrated that IFN signaling is strongly associated with treatment responses [13]. The high expression level of ISGs before treatment is a predictor of treatment failure, not only for IFN-based regimens therapy but also for DAA based therapy [13]. While the role of other immune effectors such as T cells and antibodies in cure of HCV infection has been studied in great detail [8,14–17], the role of myeloid cells is less well understood. In particular, little or no knowledge is available about the role and function of myeloid cells in the liver.

Single cell RNA-Seq (scRNA-Seq) of the liver has transformed our knowledge of liver disease biology and cancer [18]. In an elegant study, Ang Cui, Bo Li and Nadia Alatrakchi and colleagues from the Raymond T. Chung, Georg Lauer and Nir Hacohen labs at the Broad Institute of MIT and Harvard and the Massachusetts General Hospital, used scRNA-Seq to investigate the human liver myeloid cell subsets and phenotypes in 19 cured HCV-infected patients [19]. These patients of a previously described phase 3b trial were infected with HCV genotype 1a and liver fine needle aspirates (FNAs) were performed before and 12 weeks after a three-month all oral DAA regimen. Most of the patients had absent or mild fibrosis as indicated by Fibroscan analyses. By analyzing the FNAs using cell sorting with subsequent Smart-seq2 scRNA-Seq, the authors established the first single cell atlas of the myeloid compartment in the liver of chronically HCV infected patient before and after DAA treatment. Their study uses a powerful method and solid approach combining clinically relevant liver samples and the Smart-seq2 technology, which is a highly sensitive plate-based method allowing deep analysis of rare cells such as neutrophils and other granulocytes with high resolution and broad transcript coverage.

Using this state-of-the-art technology, Cui, Li et al. identified and clustered neutrophils, eosinophils, mast cells, basophils, and different subtypes of dendritic cells (DC) and monocytes/macrophages in the liver of infected and cured patients. The authors identified ISG^high^ cells in all the main myeloid cell clusters and a downregulation of ISG after DAA cure. An important new finding was the discovery of ISG^high^ neutrophil and eosinophil populations expressing high level of PDL1/L2 and of IDO respectively in HCV-infected livers, two factors involved in T cell immunosuppression. These novel results indicate that granulocytes with the strongest IFN response are likely responsible for the immunosuppressive programs in chronic HCV infection. Importantly, IFN signatures were decreased in these granulocyte populations after DAA cure in correlation with the improvement of immune responses. Another important discovery is the increase of CD1C+ proliferating dendritic cells after DAA cure with enhanced antigen presentation program via MCH-II, supporting again the restoration of chronic immune failure. Moreover, the authors unraveled 3 common gene programs shared by multiple cell types in the myeloid compartment associated with overexpression of specific marker genes: ISG and MHC-II programs were associated with the restoration of immune responses. In contrast, an alarmin gene S100 program was associated with a myeloid-derived suppressor cell-like signature which may indicate a role in immune resolution and restoration of homeostasis after virus clearance.

Finally, the authors investigated the link between ISG response and viral load. While they did not observe an association between viral load and ISG expression before DAA treatment, they showed that high viral load before DAA treatment was associated with a lower post-cure ISG expression in all the myeloid cells. These results suggest that high viral load may influence host gene expression and immune responses. In line with other studies [5–7], the authors raised the interesting hypothesis that high HCV replication may induce epigenetic modifications associated with stable expression of ISG negative regulators which could persist after cure. However, the factors regulating ISG responses are unknown and would require more investigation. By analyzing corresponding patient serum, Cui, Li et al. showed that soluble myeloid cell activation markers are still detected in some patients, even at 6-month post-DAA cure, indicating residual immune cell activation after virus clearance [19]. Their results are in line with a study recently published by Hensel et al showing that a molecular signature of T cell exhaustion is also maintained in HCV-specific CD8+ T cells even after DAA cure [8]. Together, these findings may suggest that decrease of IFN signatures in myeloid cells may only improve partially the overall immune response. Another possibility might be that the specific anti-HCV response in immune cell is different from the bystander immune cell responding to chronic inflammation.

Collectively, the liver myeloid cell atlas of HCV cure [19] is of high interest and impact by providing valuable new knowledge of the functional role of the myeloid compartment during HCV cure. A key strength of the study is the analyses of myeloid cells in the livers of infected patients using scRNA-Seq in an unbiased approach. By identifying and characterizing new myeloid cell types and associated shared immune programs involved in viral cure, the study significantly and conceptually advances our knowledge of anti-viral host immune responses during HCV elimination. It is of interest to note, that most of the patients had only mild or absent fibrosis thus only representing a subset of infected patients [19]. Although the authors did not observe a strong correlation between gene module expressions and bilirubin level or liver stiffness, further studies would be of interest to investigate whether and how fibrosis and fibrosis progressing to HCC affect the myeloid compartment and what is the role of the myeloid compartment in progression of liver disease to cancer.

Importantly, the myeloid atlas and findings of Cui, Li et al. [19] may not only be relevant for HCV cure. The discovery of novel myeloid populations and common pathways may also be exploited to target and cure other chronic viral infections of the liver, such as chronic hepatitis B or B/D. Indeed, myeloid cells have been suggested to play an important role in HBV pathogenesis [20] and immune modulation to restore dysfunctional HBV-specific immunity is one approach for new therapeutic strategies aiming at HBV cure in combination therapies [21]. Furthermore, the myeloid cell atlas and its functional conclusions will also be useful for the understanding of other immune-mediated disorders for which curative treatments are absent or unsatisfactory.

## Figures and Tables

**Figure:**
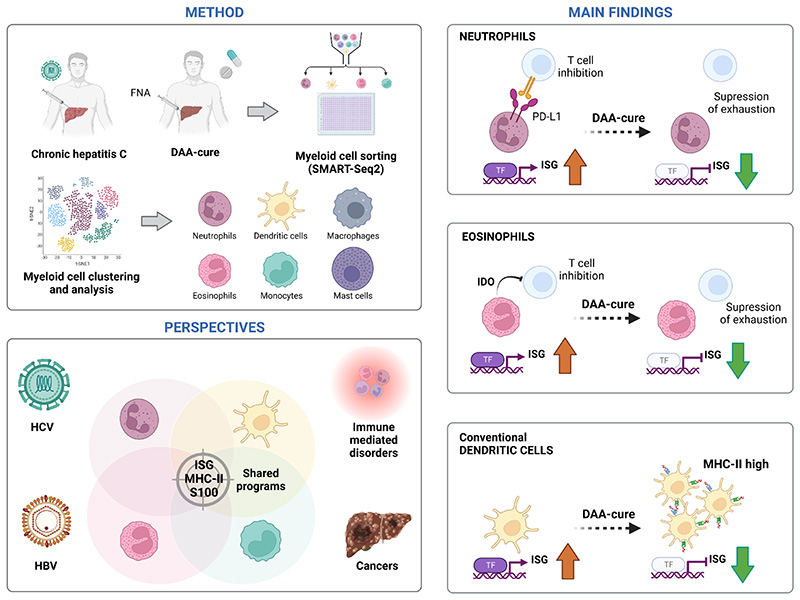
Unraveling the role of the liver myeloid compartment during hepatitis C virus cure. **Method:** Cui, Li and colleagues analyzed liver fine needle aspirates (FNAs) from HCV-infected and HCV-cured patients using cell sorting of myeloid cell populations with subsequent scRNA-Seq [19]. They identified and clustered neutrophils, eosinophils, mast cells, basophils, and different subtypes of dendritic cells (DC) and monocytes/macrophages. **Main findings**: ISG^high^ neutrophil and eosinophil populations express high level of PDL1 and of IDO respectively in HCV-infected livers, two factors involved in T cell immunosuppression. ISG expression was suppressed after DAA-cure. Proliferating conventional dendritic cells are increased after DAA cure with enhanced antigen presentation program via MCH-II. **Perspectives:** The authors unraveled shared gene programs associated with the restoration of immune responses and HCV cure. Identification of common pathways may be exploited to target and cure other chronic viral infections, auto-immune disorders and cancers. Figure was generated using Biorender.
